# Seroprevalence Survey of Avian influenza A (H5) in wild migratory birds in Yunnan Province, Southwestern China

**DOI:** 10.1186/1743-422X-11-18

**Published:** 2014-02-03

**Authors:** Hua Chang, Feiyan Dai, Zili Liu, Feizhou Yuan, Siyue Zhao, Xun Xiang, Fengcai Zou, Bangquan Zeng, YaTing Fan, Gang Duan

**Affiliations:** 1 College of Animal Science and Technology of Yunnan Agricultural University, Kunming, Yunnan 650201, China

**Keywords:** Seroprevalence, Avian influenza H5, Wild birds, Hemagglutination (HA) and hemagglutination inhibition (HI)

## Abstract

**Background:**

Highly pathogenic avian influenza virus (HPAIV) is a highly contagious disease which is a zoonotic pathogen of significant economic and public health concern. The outbreaks caused by HPAIV H5N1 of Asian origin have caused animal and human disease and mortality in several countries of Southeast Asia, such as Bangladesh, Cambodia, China, India, Indonesia, Laos, Myanmar, Thailand and Viet Nam. For the first time since 1961, this HPAIV has also caused extensive mortality in wild birds and has sparked debate of the role wild birds have played in the spread of this virus. Other than confirmed mortality events, little is known of this virus in wild birds. There is no report on the seroprevalence of avian influenza H5 infection in wild migratory birds in Yunnan Province. In this study we examined live wild birds in Yunnan Province for H5 specific antibody to better understand the occurrence of this disease in free living birds.

**Methods:**

Sera from 440 wild birds were collected from in Kunming and Northern Ailaoshan of Yunnan Province, Southwestern China, and assayed for H5 antibodies using the hemagglutination inhibition (HI) assays.

**Results:**

The investigation revealed that the seroprevalence of avian influenza H5 was as following: Ciconiiformes 2.6%, Strigiformes 13.04%, Passeriformes 20%, Cuculiformes 21.74%, Gruiformes 0%, Columbiformes 0%, Charadriiformes 0% and Coraciiformes 0%. Statistical analyses showed that there was a significant difference of prevalence between the orders (P < 0.01). Specific avian influenza H5 antibodies were detected in 23 of 440 (5.23%) sera. Mean HI titer 23 positive sera against H5 were 5.4 log_2_.

**Conclusions:**

The results of the present survey indicated that the proportion of wild birds had previously infected AIV H5 at other times of the year. To our knowledge, this is the first seroprevalence report of avian influenza H5 infection in wild migratory birds in China’ s southwestern Yunnan Province. The results of the present survey have significant public health concerns.

## Introduction

Avian influenza virus belongs to the Orthomyxoviridae family, which is divided into hemagglutinin (HA) and neuraminidase (NA) subtypes based on the cell surface antigens. There are now 18 known HA and 11 known NA subtypes when including those recently found in bats [1], H5 and H7 virus subtypes are highly virulent in poultry [2]. Avian influenza is a highly contagious disease that affects many animals, including pigs, chickens, turkey, guinea fowls, and other avian species, specially migratory water fowl [3-6]. Human had infected highly pathogenic avian influenza (H5N1) virus in Hong Kong during 1997 [7] and again in 2003 [8]. Most human cases had resulted from sporadic avian-to-human transmission of H5N1 virus during direct or close contact with sick or dead poultry [9,10].

Recent research showed that infected birds shed viruses in nasal secretions, saliva and feces [11]. Fecal-to-oral transmission is the most common mode of spread between birds [11,12]. Avian influenza outbreaks had a seasonal incidence and were usually accompanied with the season of bird migration [13] Wild birds usually act as carrier in long-distance migration, a portion of surviving birds could contribute to the potential spread of the disease [14]. It is estimated that 1.5-2.3 million birds migrate from Asia to Alaska every year, where low pathogenic H5 is endemic worldwide including the high latitudes of North America [15]. In order to better understand how these variables might influence the spatial and temporal dynamics of Avian influenza virus, Wilson et al. had reported the seroprevalence of antibodies to avian influenza viruses among wild waterfowl in Alaska, the results suggested surveillance programs include species and populations with high seroprevalences [16].

There is an important bird migrating corridor in Northern Ailaoshan in Yunnan Province, Southwestern China. Thousands of wild birds migrate from North to South in autumn every year. To our knowledge, there is little serological data concerning Avian influenza in wild birds in this migrating corridor, we decided that a serological study would be beneficial to evaluate Avian influenza (H5) exposure using the hemagglutination inhibition test.

## Results

Hemagglutination (HA) assay is detected, and the HA titer was 1:1024, the dilutions of 4HAU is 1:256. 440 blood samples were tested by HI. Twenty-three of the 440 (5.23%) wild bird serum samples were positive for H5 antibodies. Results of the investigation revealed that the seroprevalence of Avian influenza H5 was as following: Ciconiiformes 2.6%, Strigiformes 13.04%, Passeriformes 20%, Cuculiformes 21.74%, Gruiformes 0%, Columbiformes 0%, Charadriiformes 0% and Coraciiformes 0% (Table 1). The seroprevalence of Cuculiformes was highest, and the lowest seroprevalence of Gruiformes, Columbiformes, Charadriiformes and Coraciiformes was lowest. Antibody titers of 23 positive serum samples varied from 4 log_2_ to 8 log_2_ (Table 2). The highest antibody titer of four Ardeola bacchuses was 8 log_2_, the lowest antibody titer was 4 log_2_. The highest antibody titer of three Nycticorax nycticoraxs was 8 log_2_, the lowest antibody titer was 5 log_2_. The antibody titer of two Larus ridibunduses was 4 log_2_, respectively, the mean HI titer of Ciconiiformes was 5.8 log_2_. The highest antibody titer of three Otus scopses was 7 log_2_, the lowest antibody titer was 5 log_2_, the mean HI titer of Strigiformes was 5.3 log_2_. Antibody titer of Zoothera dauma was 5 log_2_. The highest antibody titer of ten Cuculus sparverioides was 7 log_2_, the lowest antibody titer was 4 log_2_, the mean HI titer of Cuculiformes was 5.1 log_2_.

**Table 1:** Serum samples of positive or negative results to AIV H5 using HI test

**Order**	**No. positive**	**Sample total**	**Positive rate (%)**
Ciconiiformes	9	346	2.6
Strigiformes	3	23	13.04
Passeriformes	1	5	20
Cuculiformes	10	46	21.74
Gruiformes	0	5	0
Columbiformes	0	2	0
Charadriiformes	0	12	0
Coraciiformes	0	1	0
Total	23	440	5.23

**Table 2:** **Distribution of layers on the basis of log**_**2 **_**HI titers obtained against AIV H5**

**Order**	**Species**	**Antibody titers using HI test**										
		**Positive samples total**	**log**_**2**_	**2 log**_**2**_	**3 log**_**2**_	**4 log**_**2**_	**5 log**_**2**_	**6 log**_**2**_	**7 log**_**2**_	**8 log**_**2**_	**9 log**_**2**_	**10 log**_**2**_
Ciconiiformes	Ardeola bacchus	9				1		1	1	1		
	Nycticorax nycticorax						1	1		1		
	Larus ridibundus					2						
Strigiformes	Otus scops	3					2	1				
Passeriformes	Zoothera dauma	1					1					
Cuculiformes	Cuculus sparverioides	10				4	2	3	1			
Total		23				7	6	6	2	2		

## Discussion

Avian influenza virus is a type A Influenza virus and zoonotic pathogen of significant economic and public health concern [2]. Some investigations showed that almost all human infected AIV H5 directly from birds, or indirectly eggs of infected birds which harborred the virus. And contaminated water, feed, insects and rodents could also act as vectors, which transmitted the disease [12,17,18]. Ailaoshan is the important passage in Northern for bird migration in Yunnan, there are thousands of wild birds through the passage. Wild birds may visit the different ponds where some domesticated birds are kept during migrating.

In addition, poultry workers in feeding poultry and butchering poultry may be associated with the infection risk [17,19]. XiangHuo [20] reported that the overall seropositive rate was 2.61% for H5N1 antibodies among poultry workers in Jiangsu, China. The wild migratory birds might be identified as a risk factor for human infection with AIV H5. Every year bird-banding was conducted by poultry workers that may be infected through contaminated bird products (i.e. feces, saliva, nasal secretions), due to their frequent exposure to birds. Facing the potential threat of avian influenza viruse H5 in wild migratory birds, it is necessary to conduct a serological survey of H5 viruses in order to better understand the status of their infection. HI test is the important test for the serological survey. Recent serological studies have been conducted to survey the seroprevalence of Avian influenza A (H5) in animals [21]. Wild birds did not inoculated vaccine, the infection status of wild birds was explored by serological survey.

In the present study, the most important finding is the finding of H5 antibody in wild birds. The overall positive rate of the wild migratory birds was 5.23% and in different orders the rates varied from 0 to 21.74%. The results were statistically analyzed by Chi-Square Tests analysis. Statistical analyses showed that there was a significant difference of prevalence between the orders (P < 0.01). In all serum samples, the highest antibody titer of serum sample was 8 log_2_, the lowest antibody titer of serum samples were 4 log_2_. The geometric mean antibody titer and seroprevalence of 23 positive serums against H5 were 5.4 log_2_ and 5.23%, respectively. Importantly, our results, in combination with different orders, suggested that the proportion of wild birds had previously infected AIV H5 at other times of the year, and inferred that wild birds may have specific patterns of AIV H5 epidemiology. In spite of presence of high antibody titers individuals birds in Cuculiformes and Ciconiiformes, no clinical symptoms were observed. Additionly, many factors could be responsible for the variability in AI H5 seroprevalence, such as species, ages, geographic locations, and years. Our study examined only seroprevalence of AI H5 antibodies across a range of wild migratory species in Northern Ailaoshan in Yunnan. In order to better understand how these variables might influence the spatial and temporal dynamics of AI H5 in wild birds, further analysis and surveillance are needed to adequately validate our results. The seroprevalence of the free living birds were only monitored in this study, our study cannot rule out the possibility of cross reaction with low pathogenic avian influenza virus H5, future studies should use an H5 antigen with a different neuraminidase. In addition, some other studies had reported that there have been numerous wild birds mortality events from the HPAIV H5N1 [22,23], but some reports concerned that apparently healthy wild birds infected with HPAI H5N1may distribute this virus during migration [22,24]. In order to better understand the occurrence of this disease in wild birds in Yunnan, the seroprevalence of H5 avian influenza surveillance programs must be performed, and some laboratories tests detect suspected seropositive samples against at least two different antigens with different neuraminidase subtypes.

## Conclusions

To our knowledge, this is believed to be the first seroprevalence report of avian influenza virus (H5) infection of the wild migratory birds in Yunan Province, China. In the present survey, the positive rate of the wild migratory birds we found was 5.23%. Statistical analyses showed that there was a significant difference of prevalence between the orders (P < 0.01). The results indicated that the proportion of wild birds had previously infected AIV H5 at other times of the year, which raises significant public health concerns. Therefore, it is necessary for poultry workers, public health authorities, and tourists to pay more attention to this problem.

## Materials and methods

### Ethics statement

The present study was approved by Wildlife Conservation and Nature Reserve of Forestry Department in Yunnan province. All sampling (i.e., serum collection) was accomplished according to the Animal Ethics Procedures and Guidelines of the People’s Republic of China. All birds were captured, sampled and subsequently released in the wild.

### The investigated regions

Yunnan Province is known for its richness in natural resources, covering approximately 394,000 square kilometers in Southwestern China. Positive swab samples of H5N1 avian influenza in poulty and wild birds were detected in in boundary regions of Yunnan province [25], but there is no report on the seroprevalence of avian influenza H5 infection in wild migratory birds in Yunnan Province at present. The survey was conducted in Kunming (latitude 102.73°, longitude 25.04°) and Ailaoshan (latitude 102.52°, longitude 24.35°), Ailaoshan is located in Yuxi City, where is an important bird migrating corridor in Northern Ailaoshan. Bird-banding is one of important approaches to study migrating birds every year, there were thousands of migratory birds.

### Samples and data collection

During October-November 2011, we collected serum samples from 440 wild migratory birds showing no obvious signs of disease in this serological study. These serum samples were classified as eight orders (Strigiformes, Ciconiiformes, Passeriformes, Gruiformes, Columbiformes, Charadriiformes, Coraciiformes, Cuculiformes), twelve family, and twenty-four species (Table 3).

**Table 3:** Classification of serum samples

**Order/total**	**Family**	**Species**	**Number**	
Cuculiformes	Cuculidae	Cuculus sparverioides	36	
		Cuculus poliocephalus	2	
		Cuculus canorus	2	
		Cuculus saturatus	1	
		Clamator coromandus	1	
		Common Hawk-Cuckoo	1	
		Eudynamys scolopacea	1	
	Centropodidae	Centropus bengalensis	2	
Strigiformes	Strigidae	Otus scops	22	
		Otus spilocephalus	1	
Ciconiiformes	Ardeidae	Ardeola bacchus	27	
		Bubulcus ibis	3	
		Nycticorax nycticorax	12	
	Laridae	Larus ridibundus	304	
Passeriformes	Muscicapidae	Zoothera dauma	2	
		Myophonus caeruleus	1	
		Niltava banyumas	1	
	Dicruridae	Dicrurus hottentottus	1	
Gruiformes	Rallidae	Amaurornis phoenicurus	1	
		Gallinula chloropus	4	
Columbiformes	Columbidae	Treron sphenura	2	
Charadriiformes	Charadriidae	Vanellus cinereus	8	
	Scolopacidae	Scolopax rusticola	4	
Coraciiformes	Alcedinidae	Halcyon pileata	1	
Total	8	12	24	440

Wild birds were captured, and each bird was collected up to 2 ml of blood, in total, 440 blood samples were obtained from the wing vein of wild birds. After clotting and centrifugalization, serum was separated at 3000 rpm for up to 10 min and stored at -20°C until further analysis. All tubes were labeled with date and species.

### Hemagglutination (HA) and Hemagglutination Inhibition (HI) assays

The serological technique used was Hemagglutination (HA) and Hemagglutination Inhibition (HI). H5 antigen was purchased from Harbin Veterinary Research Institute, China. 1% specific pathogen free chicken red blood cells were prepared. HA titers were determined using 25 ul of 1% chicken red blood cells in PBS to 25 ul of a two-fold serial dilution of virus in 96 ‘V’-well microtiter plates. Microtiter plates were incubated for 30 minutes at 30°C. HA titers were subsequently calculated as the reciprocal value of the highest dilution that caused complete hemagglutination. The HI assay was performed using doubling dilution in PBS, 1% chicken red blood cells, and 4 hemagglutinating units of H5 antigen. After 45 min at 25°C, 25 ul 1% red blood cells was added and the final result was read after 50 min.

### Criteria for seropositivity

HI titers were calculated as the reciprocal of the highest serum dilution that inhibited 4HA antigen. When HI titer of negative serum was no more than 2 log_2_, the test could be established. If HI titer of the tested serum was less than or equal to 3 log_2_, it will be negative; if HI titer is equal to 4 log_2_, it will be suspicious (suspicious samples should be tested in duplicate tests, if HI titer is more than or equal to 4 log_2_, it will be positive); if HI titer is less than or equal to 3 log_2_, it will be negative; if HI titer is equal to or more than 5 log_2_, it will be positive.

### Statistical analysis

Differences in seroprevalence of infected birds were analyzed using Chi-Square tests in SPSS (Release18.0 standard version) for Windows. A probability (P) value < 0.05 was considered as statistically significant between levels within factors and interactions.

## Competing interests

The authors declare no conflict of interest.

## Authors’ contributions

HC carried out most of the experiments and wrote the manuscript. FYD, ZLL, FZY and SYZ performed the experiments. GD, FCZ, BQZ, YTF and XX analyzed the data. All authors read and approved the final manuscript.

## Authors’ information

Hua Chang, Feiyan Dai, Zili Liu, and Feizhou Yuan are considered as first authors.
